# Single Channel Recordings Reveal Differential β2 Subunit Modulations Between Mammalian and *Drosophila* BK_Ca_(β2) Channels

**DOI:** 10.1371/journal.pone.0163308

**Published:** 2016-10-18

**Authors:** Zhenzhen Yan, Bin Hu, Zhigang Huang, Ling Zhong, Xiying Guo, Anxi Weng, Feng Xiao, Wenping Zeng, Yan Zhang, Jiuping Ding, Panpan Hou

**Affiliations:** 1 Key Laboratory of Molecular Biophysics of the Ministry of Education, College of Life Science and Technology, Huazhong University of Science and Technology, Wuhan, Hubei, 430074, China; 2 Department of Biomedical Engineering, Center for the Investigation of Membrane Excitability Diseases, Washington University in St Louis, St Louis, 63130, United States; 3 Key Laboratory of Image Processing and Intelligent Control, Huazhong University of Science and Technology, Ministry of Education, Department of Biomedical Engineering, College of Life Science and Technology, Wuhan, Hubei, China; Xuzhou Medical College, CHINA

## Abstract

Large-conductance Ca^2+^- and voltage-activated potassium (BK) channels are widely expressed in tissues. As a voltage and calcium sensor, BK channels play significant roles in regulating the action potential frequency, neurotransmitter release, and smooth muscle contraction. After associating with the auxiliary β2 subunit, mammalian BK(β2) channels (mouse or human Slo1/β2) exhibit enhanced activation and complete inactivation. However, how the β2 subunit modulates the *Drosophila* Slo1 channel remains elusive. In this study, by comparing the different functional effects on heterogeneous BK(β2) channel, we found that *Drosophila* Slo1/β2 channel exhibits “paralyzed”-like and incomplete inactivation as well as slow activation. Further, we determined three different modulations between mammalian and *Drosophila* BK(β2) channels: 1) dSlo1/β2 doesn’t have complete inactivation. 2) β2(K33,R34,K35) delays the dSlo1/Δ3-β2 channel activation. 3) dSlo1/β2 channel has enhanced pre-inactivation than mSlo1/β2 channel. The results in our study provide insights into the different modulations of β2 subunit between mammalian and *Drosophila* Slo1/β2 channels and structural basis underlie the activation and pre-inactivation of other BK(β) complexes.

## Introduction

Large-conductance Ca^2+^- and voltage-activated potassium (MaxiK or BK) channels are widely distributed in mammalian tissues [1]. As a voltage and calcium sensor [2], the open probability of BK channel increases with both membrane depolarization and increased intracellular calcium concentration [3–7], thereby playing a critical role in modulating many significant physiological activities, including neurotransmitter release, endocrine secretion in neurons or endocrine cells, contraction of smooth muscle cells, frequency tuning in hair cells, and pain signaling inhibition in DRG neurons [3–13].

The BK channel is a tetramer with four pore-forming α subunits, which is encoded by the gene *Slo1* (KCNMA1) [14, 15]. Each α subunit has seven transmembrane segments S0–S6 with an extracellular N-terminus and a large cytoplasmic C-terminus, which contains a mechanical linker (C-linker) [16] and two Rossmann-fold Regulator of Conductance of K^+^ (RCK) domains with two Ca^2+^ binding sites (calcium bowl and Slo1 (D367, E535)) that are required for activation by calcium [2, 17–20]. A passive spring model was proposed as the mechanism whereby the length of the C-linker influenced the BK channel activation and gating [16]. The C-linker was also found to be critical for neuronal excitability and BK opener binding [21, 22]. The N-terminus of the RCK1 domain is a region that includes the secondary structures βA-αC, and it was designated as the AC region [23, 24]. The AC region is important for Ca^2+^ binding and gating, Mg^2+^ binding, epilepsy-associated Ca^2+^ sensing potentiation, and the binding of drugs, such as PIP2, H^+^, CO, and ethanol [24–32]. As a unique structure located between the RCK domain and the pore domain (PD) [33], it is possible that the C-linker and AC region are responsible for transferring the mechanical Ca^2+^ gating force from the RCK domain to the PD.

The properties of native BK channels are diversified by associating with tissue-specific auxiliary β1-β4 subunits [34–36]. One BK channel can associate with up to four auxiliary β subunits at 1:1 stoichiometry with Slo1α subunits [37, 38]. These auxiliary subunits share similar topologies each having two transmembrane (TM1 and TM2) segments, intracellular N and C terminals, and a large extracellular loop [37–41]. The β1 and β2 subunits induce increased apparent Ca^2+^ sensitivity and slowing of the macroscopic kinetics [35, 42, 43]. The β2 subunit also induces fast and complete N-type inactivation [39, 40, 44, 45]. The inactivation domain (ID) of the β2 NH2-terminus, which comprises a hydrophobic head group Phe–Ile–Trp (FIW), blocks the ion-conducting pore at the cytoplasmic mouth of BK channels, thereby leading to N-type inactivation via a two-step inactivation process [46, 47].

Evidence suggests that the modulation of BK-type channel gating by β2 subunits involves both the transmembrane and C linker-AC region of Slo1 [48]. Our previous studies demonstrated that the dominant roles of regulation via augmented Ca^2+^ sensitivity and the pre-inactivation properties were due to pairwise interactions between the N-terminus of the β2 subunit and the C-linker and AC region of the Slo1α subunit [49, 50].

When studying the gating property, β subunit modulation, and pharmacology of BK channels, it is common to compare the functional differences between mammalian (mouse or human) Slo1/β and *Drosophila* dSlo1/β channels due to the “insulation” property of dSlo1 in certain respects. For instance, both β1 and β2 subunits do not enhance activation of dSlo1 in the same manner as the hSlo1 channel [48, 51]. Thus, by comparing the functional differences between mammalian Slo1/β2 and *Drosophila* Slo1/β2 channels, researchers found that the modulation of BK channel gating by β2 subunits involves both the membrane-spanning and cytoplasmic domains of mSlo1 [48]. In addition, dSlo1 does not respond to omega-3 docosahexaenoic acid (DHA) in the same manner as the hSlo1 channel, and study showed that a point mutation in the hSlo1 channel impaired its sensitivity to DHA [52]. However, how does β2 subunit modulate the *Drosophila* Slo1 channel remains elusive.

Based on the differential modulation properties between dSlo1/β2 and mammalian Slo1/β2 channels, in this study, we found that the “inactivation ball” FIW was not enough for complete inactivation of dSlo1/β2 channel, and may serve as a retention signal for dSlo1/β2 but not for mammalian Slo1/β2. And the positively charged residues β2(K33,R34,K35) led to slow activation in dSlo1/Δ3-β2, but not in mammalian Slo1/Δ3-β2. Furthermore, we demonstrated that dSlo1/β2 channel had enhanced pre-inactivation than mSlo1/β2 channel. The results in our study provided insights into the different modulations of β2 subunit between mammalian and *Drosophila* Slo1/β2 channels and structural basis underlie the activation and pre-inactivation of other BK(β) complexes.

## Results

### dSlo1/β2 channel doesn’t have complete inactivation

**Fig 1:**
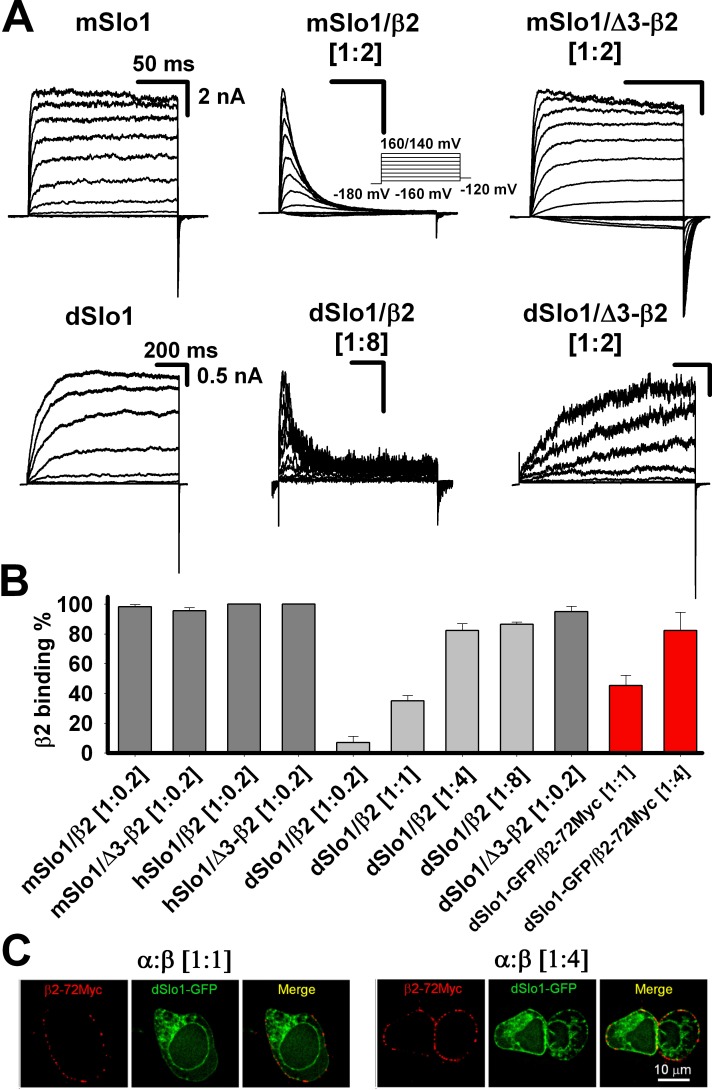
dSlo1/β2 channel doesn’t have complete inactivation. **(A)** Macroscopic currents of mSlo1, mSlo1/β2, mSlo1/Δ3-β2, dSlo1, dSlo1/β2, and dSlo1/Δ3-β2 obtained from inside-out patches in the presence of 10 μM Ca^2+^ according to the protocol indicated. Scale bars represent 50 ms and 2 nA for mSlo1/x channels, and 200 ms and 0.5 nA for dSlo1/x channels. The α:β transfection ratio was 1:8 for dSlo1/ β2, and 1:0.2 for other combinations. The voltage ranges of the test potential were from -160 mV to +160 mV for mSlo1, mSlo1/β2, and mSlo1/Δ3-β2, and from -160 mV to 140 mV for dSlo1, dSlo1/β2 and dSlo1/Δ3-β2. **(B)** β2 binding percentages of a series of α/β2 channels at different α:β transfection ratios. Gray bars were obtained from patch clamp recordings and the red bars from immunofluorescence imaging. The mSlo1/β2 channel currents were analyzed based on patches with entire inactivation, mSlo1/Δ3-β2 based on the G-V relationship left shift, dSlo1/β2 based on inactivation or briefly opening recordings, and dSlo1/Δ3-β2 based on slow activation. dSlo1-GFP/β2-72Myc at [1:1] (N = 60) and at [1:4] (N = 61) were analyzed based on surface immunofluorescence images of β2-72Myc and then normalized against the dSlo1/β2 [1:4] results obtained from patch clamp recordings. The error bars represent the standard error of the mean (SEM). **(C)** Immunofluorescence imaging of dSlo1-GFP/β2-72Myc at different α:β transfection ratios of 1:1 (left) and 1:4 (right). Scale bar represents 10 μm.

When studying the modulation of Slo1 by β2 subunit, it is important to saturate the β2 expression to get stable results. Our previous study has demonstrated that in human embryonic kidney (HEK) 293 cell expression system, the α:β2 transfection ratio at 1:0.2 was good enough to maintain saturate β2 binding for every mSlo1/β2 channel. For instance, when the transfection ratio of α:β2 was 1:0.2, all of the random tests of mSlo1/β2 channel currents exhibited rapid and complete inactivation, which indicated that the binding rate between α and β2 was about 100% (Fig 1A and 1B), and at the same 1:0.2 ratio in the mSlo1/Δ3-β2 channel (the inactivation ball FIW was deleted), the V_50_ of all the random tests had obvious left shifts to −30 mV from approximately +25 mV (mSlo1) in 10 μM Ca^2+^, which suggested that the binding rate between α and β2 was also approximately 100% (Fig 1B) [49, 50]. hSlo1/β2 and hSlo1/Δ3-β2 shared the same results (Fig 1B). However, in the dSlo1/β2 channel, when the transfection ratio of α:β2 was 1:0.2, only a few (about 7%) random tests exhibited inactivation or brief openings (Fig 1B), thereby indicating that the binding rate between dSlo1 and β2 was much lower than that in the mSlo1/β2 channel.

To test this hypothesis, we increased the β2 transfection amount (α:β2) to 1:1, 1:4, and 1:8. As the number of transfected β2 subunits increased, we obtained more examples with inactivation or brief openings, and when the transfection ratio of dSlo1:β2 was 1:4, the β2 binding ratio was almost saturated at 85%. However, for dSlo1:Δ3-β2, all of the random tests exhibited slow activation even when the transfection ratio was 1:0.2, which suggested that the β2 binding rate was again about 100% (Fig 1B). These results indicate that the inactivation ball FIW in the dSlo1/β2 channel might serve as a retention signal that could block the binding of β2. All statistical results were listed in Fig 1B.

We further confirmed the electrophysiology results by immunofluorescence imaging. Green fluorescent protein (GFP, green) and Myc (EQKLISEEDL, red) were inserted into the C-terminal of dSlo1 and the extracellular loop of β2 subunit, respectively, as fluorescent tags to facilitate our fluorescent analyses. The fused fluorescent proteins did not alter the gating properties of the wild-type (WT) channels (S1 Fig). According to the results of electrophysiological experiments, we selected two representative dSlo1-GFP:β2-72Myc transfection ratios of 1:1 and 1:4, and measured the membrane fluorescence intensities of β2-72Myc (Fig 1B and 1C). After normalizing the fluorescent intensity of dSlo1-GFP/β2-72Myc [1:4] against the electrophysiology result for dSlo1/β2 [1:4], there was no significant difference between the fluorescent intensity of dSlo1-GFP/β2-72Myc [1:1] and the electrophysiology result for dSlo1/β2 [1:1] (Fig 1B). These results suggested that the FIW was a retention signal in the Drosophila Slo1/β2 channel, but not in mammalian Slo1/β2 channels.

### β2(K33E,R34D,K35E) generated slow activation in dSlo1/Δ3-β2 channel, but not in mSlo1/Δ3-β2 channels

**Fig 2:**
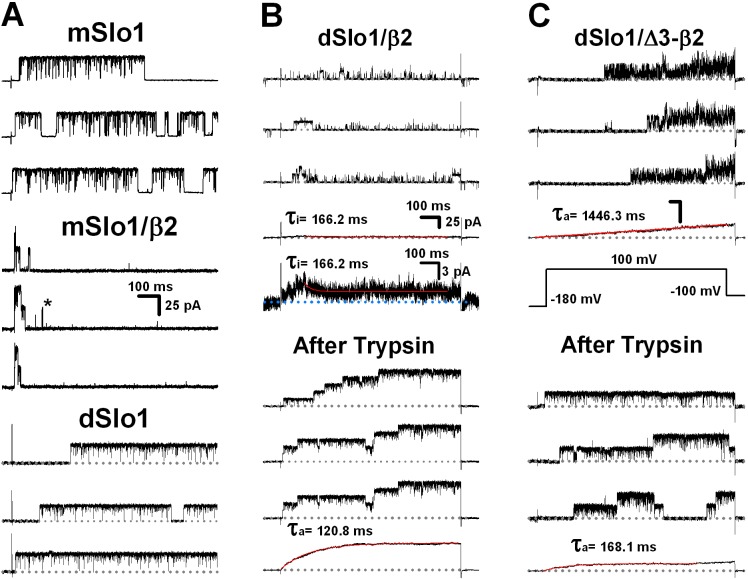
Single channel recordings of mSlo1 and dSlo1 combinations with β2 subunit. **(A)** Representative single channel recordings of mSlo1, mSlo1/β2, and dSlo1 channels from inside-out patches in the presence of 10 μM Ca^2+^ using a depolarizing voltage of 100 mV, as indicated in (B). The scale bars were 100 ms and 25 pA, respectively. **(B)** Representative single channel recordings of the dSlo1/β2 channel before (top) and after (bottom) applying 0.2 mg/ml trypsin. The bottom black traces are ensemble averages from 100 consecutive sweeps and its enlarged trace. Red traces are single exponential fits to the black traces. The inactivation τ_i_ and activation time τ_a_ constants were 166.2 ms and 120.8 ms, respectively. The α:β transfection ratio was 1:8. The scale bars were shown as indicated. **(C)** Representative single channel recordings of the dSlo1/Δ3-β2 channel before (top) and after (bottom) applying 0.2 mg/ml trypsin. The bottom black traces are ensemble averages from 100 consecutive sweeps. Red traces are single exponential fits to the black traces. The activation time constants τ_a_ were 1446.3 ms and 168.1 ms, respectively. The α:β transfection ratio was 1:8. The scale bars were 100 ms and 25 pA as shown in (B).

It was interesting to notice that compared with WT dSlo1 channel currents, the currents of dSlo1/β2 channel was much smaller in amplitude and filled with “paralyzed”-like brief openings (Fig 1A). After deleting the inactivation ball FIW of β2 subunit, the dSlo1/Δ3-β2 channel exhibited dramatically slower activation (Fig 1A). To elucidate the underlying behavior of the channels, we performed single channel recordings of different mSlo1 and dSlo1 channel combinations (Fig 2). The currents were recorded with a Ca^2+^ concentration of 10 μM in inside-out mode at +100 mV as indicated. A -180 mV (100 ms) pre-pulse was used to eliminate the possibility of inactivation [49]. Fig 2B showed the single-channel recordings of dSlo1/β2. Unlike the mSlo1/β2 channel, which exhibited fast and complete inactivation **(**τ_i_ **=** 15.1 ms), mainly long openings and rare brief openings (Fig 2A “*”), dSlo1/β2 exhibited slower inactivation **(**τ_i_ **=** 166.2 ms) and much more brief openings, as well as a detectable steady-state current after an average of 100 consecutive single-channel traces, which were consistent with the macroscopic currents shown in Fig 1A. However, after the application of 0.2 mg/ml trypsin [47], the brief openings and inactivation were cut off gradually, whereas the current amplitude and opening time duration were greatly increased, thereby exhibiting similar characteristics to the dSlo1 alone channel (Fig 2A and 2B). This result indicated that the “paralyzed”-like brief openings came from the N-terminal of β2 since trypsin could digest the N-terminal of β2 [47, 49].

Then we confirmed the slow activation of dSlo1/Δ3-β2 at single channel level. The dSlo1/Δ3-β2 single channel currents exhibited obvious hysteresis in terms of the activation rate **(**τ_a_ = 1446.3 ms) and the burst openings made the channel open in an unusual manner so it appeared “paralyzed” as well (Fig 2C). After applying 0.2 mg/ml trypsin, the burst openings and slow activation were also eliminated gradually, and the current eventually appeared to be more similar to that of dSlo1 alone (Fig 2A and 2C). These results suggested that the slow activation might also have been attributable to the N-terminal of β2.

**Fig 3:**
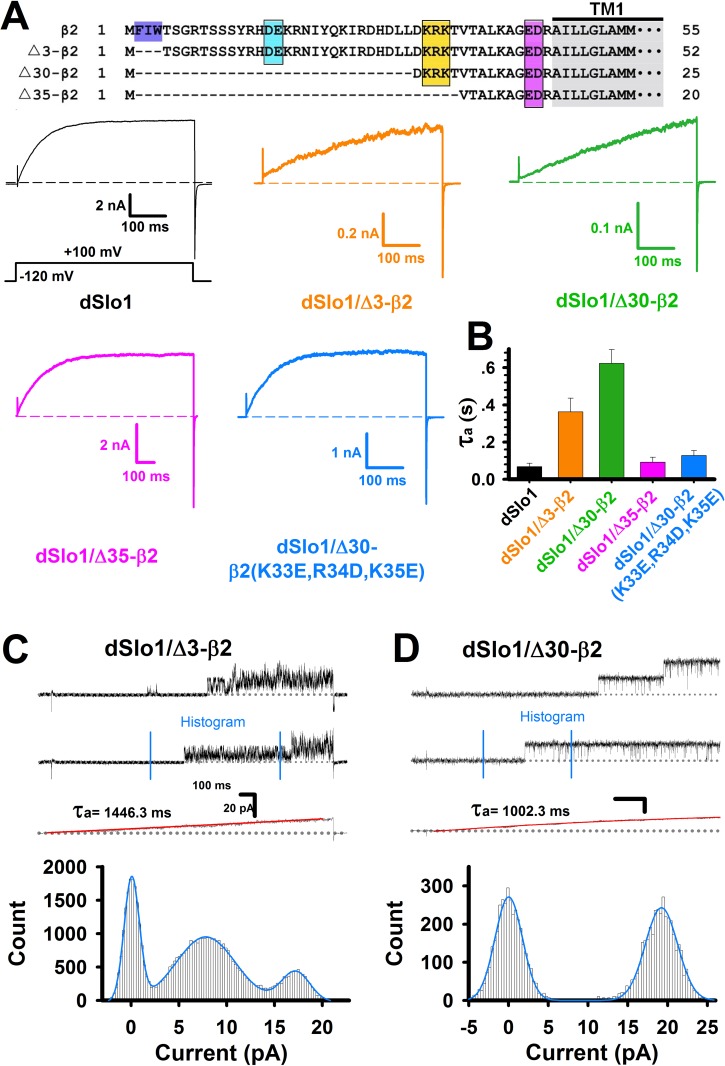
β2(K33,R34,K35) caused slow activation in dSlo1/Δ3-β2 channel. **(A)** Top panel was the construct for β2 subunit truncations Δ3-β2, Δ30-β2, and Δ35-β2. Bottom panel showed the traces recorded at 100 mV in 10 μM Ca^2+^ solution for dSlo1 (black), dSlo1/Δ3-β2 (orange), dSlo1/Δ30-β2 (dark green), dSlo1/Δ35-β2 (pink), and dSlo1/Δ30-β2(K33,R34,K35) (light blue). **(B)** Average activation time constants (τ_a_) for dSlo1 (68.55 ± 6.65 ms), dSlo1/Δ3-β2 (288.05 ± 74.01 ms), dSlo1/Δ30-β2 (623.37 ± 74.18 ms), dSlo1/Δ35-β2 (92.48 ± 25.88 ms), and dSlo1/Δ30-β2(K33,R34,K35) (127.92 ± 26.22 ms). Traces were fitted with a single exponential equation. **(C–D)** Single channel recordings and current histograms for the dSlo1/Δ3-β2 and dSlo1/Δ30-β2 channels. The currents between the blue lines were the raw data used for histogram analysis and the histogram were fitted with triple or double Gaussian equations.

To precisely determine the key residues for the slow activation, we systematically shortened the length of β2 N-terminal and then tested their effects on the slow activation (Fig 3). The macroscopic currents of dSlo1 with different β2 N-terminus lengths were shown in Fig 3A. After truncating the first 3 or 30 amino acids from the N-terminus of β2, dSlo1/Δ3-β2 and dSlo1/Δ30-β2 both clearly exhibited slow activation, but when the first 35 amino acids were cut off, the dSlo1/Δ35-β2 activation time constant was obviously accelerated to the level of dSlo1 channel activation (Fig 3A and 3B), which suggested that the amino acids β2(D32,K33,R34,K35,T36) located at positions 32–36 of the N-terminus of β2 played a key role in generating the slow activation. The three positively charged amino acids β2(K33,R34,K35) were mainly considered. When they were mutated to negative charges, dSlo1/Δ30-β2(K33E,R34D,K35E) exhibited the same activation rate as the dSlo1/Δ35-β2 channel (Fig 3A and 3B), thereby indicating that β2(K33,R34,K35) was responsible for the slow activation. To exclude the possibility that Δ30-β2(K33E,R34D,K35E) did not express, we tested the dSlo1/β2(K33E,R34D,K35E) currents. Although we didn’t see clear inactivation, the currents also showed “paralyzed”-like brief openings and the amplitude was much smaller than the dSlo1 control currents from the same bench of cells (S2 Fig), which demonstrated that β2(K33E,R34D,K35E) did express well.

Single channel analysis of dSlo1/Δ3-β2 burst openings indicated that even if the inactivation ball FIW was deleted from β2, the remaining inactivation chain could still enter the channel pore to block the channel opening by half (Fig 3C). When the first 30 amino acids were cut from the N-terminal of β2, the chain length might not be sufficiently long to enter the pore, so the single channel recordings indicated long openings as normal dSlo1 but the slow activation remained (Fig 3D) since the β2(K33E,R34D,K35E) was still working.

A comprehensive macroscopic activation or inactivation currents were also performed to confirm the time constants of the averaged single channel recordings (S3 Fig).

### Enhanced pre-inactivation in dSlo1/β2 channel

**Fig 4:**
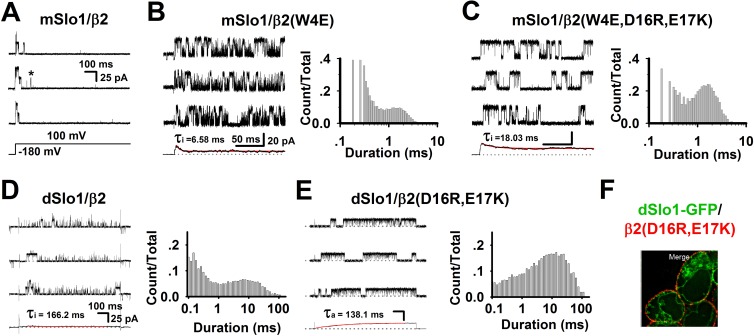
Single channel recordings revealed the enhanced pre-inactivation in dSlo1/β2 channel. **(A)** Representative single channel recordings of mSlo1/β2 from inside-out patches in the presence of 10 μM Ca^2+^ using a depolarizing voltage of 100 mV as indicated. The scale bars were 100 ms and 25 pA, respectively. **(B-C)** Three representative single-channel traces of mSlo1/β2(W4E) and mSlo1/β2(W4E,D16R,E17K) recorded from inside-out patches at +100 mV in 10 μM Ca^2+^. Bottom traces showed ensemble averages of 100 consecutive sweeps. Initial capacitive currents within the first 0.5 ms of voltage steps were deleted. Right, histogram of open-time durations from the two single-channel recordings. **(D-E)** Three representative single-channel traces of dSlo1/β2 and dSlo1/β2(D16R,E17K) recorded from inside-out patches at +100 mV in 10 μM Ca^2+^. Bottom traces showed ensemble averages of 100 consecutive sweeps. Right, histogram of open-time durations from the two single-channel recordings. **(F)** Immunofluorescence imaging of dSlo1-GFP/β2(D16R,E17K)-72Myc at different α:β transfection ratios of 1:4.

In previous studies, we demonstrated the two-step inactivation process for the mSlo1/β2 channel: C↔O↔O*↔I [49]. During inactivation, the inactivation ball FIW swung randomly with the inactivation chain [45]. After the channel opened, two negatively charged residues located in the inactivation chain at β2(D16,E17) and two positively charged residues located in the intracellular mouth of the pore at mSlo1(K330,K331) would combine to form a pre-inactivation site (PI site, O* state), after which the inactivation ball swung rapidly into the channel pore to block the conducting pathway and inactivate the channel. Hence, according to the opening time duration during the single channel behaviors, there were two distinct open states, i.e., relatively long opening (O state) and short-lived brief opening (O* state) [49]. In wild-type mSlo1/β2 channel, this brief opening happened rarely [47, 49]. Occasionally, however, some brief openings were evident in our single channel recordings containing several mSlo1/β2 channels (Fig 4A, “*”). A mutation β2(W4E) was used to test the existence of the pre-inactivated state (O*) of mSlo1/β2 based on the enhanced probability of brief openings in the steady-state compared with mSlo1/β2 (Fig 4A and 4B) [47, 49]. And after we destroyed the PI site by mutating the two negatively charged residues on N terminal β2 to positively charged residues, mSlo1/β2(W4E,D16R,E17K) displayed only long openings (Fig 4C).

Thus, we hypothesized that the brief openings seen in the single channel currents of dSlo1/β2, which were similar to the mSlo1/β2(W4E) channel currents, were attributable to the pre-inactivation of the channels. Destruction of the PI site by trypsin could eliminate the brief openings of the dSlo1/β2 channel supported this idea (Fig 2B), and again, we performed single channel recordings of dSlo1/β2(D16R,E17K), which would destroy the PI site as well. The currents showed only long openings similar as dSlo1 channel currents (Figs 2 and 4E) as predicted. To confirm that β2(D16R,E17K) was expressed in the single channel currents, we further tested immunofluorescence imaging of dSlo1-GFP/β2(D16R,E17K)-72Myc (Fig 4F). Taken together, our results demonstrated that the pre-inactivation property was not only conserved in dSlo1/β2 channel, but also enhanced without the enhancement of β2(W4E) (Figs 2B and 4D).

## Discussion

The β2 subunit diversifies the properties of the Slo1 α subunit, which raises many interesting and significant questions. Comparing the functional differences between homologous BK channels is a simple method for studying the BK channel gating, βx modulation, and pharmacological properties of the channel [24, 48, 52]. Using the xSlo1/β2 channel as a tool, in this study, we discovered three interesting functional differences regulated by β2 subunit between mammalian (mouse or human) and *Drosophila* Slo1/β2 channels.

Unlike the β1 subunit, which could be expressed freely on the membrane [53], a four-turn α-helical segment in the N-terminal of β2 subunit was shown to serve as a retention signal that could prevent surface expression of the β2 subunit [54]. When we doubled the inactivation ball FIW (double FIW or dFIW), “dFIW-β2” was retained firmly in the endoplasmic reticulum even when it was co-expressed with the mSlo1 α subunit and the BK currents were eliminated from the plasma membrane, which suggests that dFIW was an enhanced retention signal for the mSlo1/β2 complex [49]. However, for dSlo1/β2 channel, it appeared that FIW itself was a retention signal and dSlo1 and β2 binding (or interactions) on the plasma membrane could be adjusted by changing the amount of transfected β2 (Fig 1B and 1C). The detailed reason might also be related to the hydrophobicity of FIW.

The slow activation came from the three positively charged residues β2(K33,R34,K35) resided in the N terminal of β2 subunit (Figs 2 and 3). However, understanding why the lack of FIW dSlo1/Δ3-β2 currents led to a dramatically slower activation rate requires further research. It is interesting that a BK channel can be activated so slowly at a Ca^2+^ concentration of 10 μM **(**τ_a_ ~ 1 s; Figs 2 and 3), and this may provide a more distinctive model for studying the Ca^2+^ and voltage gating processes in the BK channel. A more precise interpretation of these behaviors may depend on the structure of the BK(β2) channel complex. For the enhanced pre-inactivation of dSlo1/β2 channel, it was reasonable to believe that the enhancement might be attributed to the different C-linker and AC regions between mammalian and *Drosophila* Slo1 channel. Based on the crystal structures of the BK channel gating ring (RCK domains) in both the open and closed states, as well as the recently reported cryo-electron microscopy structure of the BK(Slo2.2) channel, the C-linker and AC region are located closest to the transmembrane domain [33, 55–57] (S4 Fig). As a unique part of the Ca^2+^ gating pathway, because they are located between the Ca^2+^ binding RCK domain and channel pore opening domain, the C-linker and AC region are inevitably responsible for transferring the Ca^2+^ gating force from the RCK domain to the channel pore [16, 24, 25]. Regarding the BK channel microstructure, any differences in the amino acid sequences will lead to variations in structure and thus the channel functions, especially when the different residues are charged or hydrophobic amino acids because this may allow new possibilities for the formation of electrostatic or hydrophobic interactions. Thus, multiple BK channel functions could be affected or even changed by electrostatic or hydrophobic interactions, such as the voltage dependence [58, 59], single-channel conductance [60, 61], gating dynamics [62–65], rectification characteristics [60, 66, 67], drug sensitivity [67–69], inactivation property [49], and membrane expression [49, 54].

**Fig 5:**
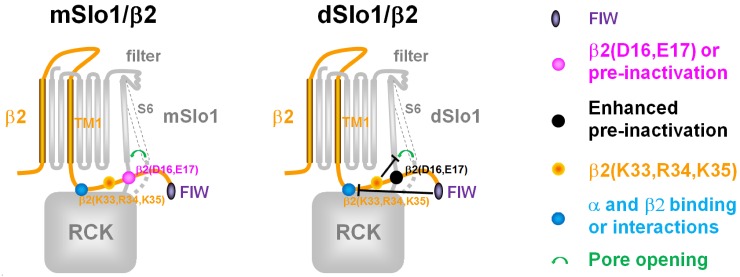
Cartoon schematic to clarify the different modulations of β2 subunit between mammalian and *Drosophila* BK_Ca_(β2) channels. Gray and orange bars indicated dSlo1 or mSlo1 and β2 subunit, respectively. Blue, orange, and pink circles indicated the α and β2 binding or interactions, β2(K33,R34,K35), and β2(D16,E17) or pre-inactivation, respectively. The black circle indicated the enhanced pre-inactivation in dSlo1/β2 channel. Firstly, in dSlo1/β2 channel, the inactivation ball FIW is not enough for the complete inactivation, and may suppress the α and β2 binding or interactions. Secondly, β2(K33,R34,K35) delays the pore opening (or voltage sensor movement). Thirdly, dSlo1/β2 channel has enhanced pre-inactivation than mSlo1/β2 channel.

The pre-inactivation site was formed by the interaction between mSlo1(K330,K331) and β2(D16,E17). In mammalian mSlo1 or hSlo1, there was two positively charged lysine residues at positions (K330,K331). By contrast, the corresponding position of *Drosophila* Slo1, dSlo1(N327,K328), had only one positively charged residue. The single “arm” at dSlo1(N327,K328) may lack the ability to capture β2(D16,E17). Instead, β2(D16,E17) might interact with other positively charged residues within C-linker and AC region such as dSlo1(K334,R335) or dSlo1(R377,K378) to form a “misplaced” PI site, thereby resulting in “paralyzed” inactivation (enhanced pre-inactivation) at either the single channel or macro-current level (Figs 1, 2 and 3). The “paralyzed” single channel recordings obtained for dSlo1/β2 also indicated that the pre-inactivation state (O*) was enhanced without the help of β2(W4E) (Fig 4), so FIW might not interact as strongly with the pore as that found in the mammalian mSlo1 and the hSlo1 channel. Therefore, the mammalian (mouse and human) Slo1 channels and *Drosophila* Slo1 could have different pore structures. When FIW was removed from β2, the remaining inactivation chain could still enter the pore to reduce the channel opening by half (Fig 3). This model can be used as a tool to study the pore structure or size of the BK channel, as well as the interaction between the β2 N-terminal and pore region [46].Finally, we made a cartoon schematic to clarify the three different modulations of β2 subunit between mammalian and *Drosophila* BK_Ca_(β2) channels (Fig 5). Gray and orange bars indicated dSlo1 or mSlo1 and β2 subunit, respectively. Blue, orange, and pink circles indicated the α and β2 binding or interactions, β2(K33,R34,K35), and β2(D16,E17) or pre-inactivation, respectively. Firstly, in dSlo1/β2 channel, the inactivation ball FIW is not enough for complete inactivation of dSlo1, and resulted in a much lower binding rate than mSlo1/β2 channel at the same α:β2 transfection ratio (Fig 1). Secondly, β2(K33,R34,K35) delayed the channel activation (pore opening, or voltage sensor movement, or the VSD-PD coupling), the channel currents exhibited obvious hysteresis in terms of the activation rate (Figs 1, 2 and 3). Thirdly, dSlo1/β2 channel had enhanced pre-inactivation than mSlo1/β2 channel without the help of β2(W4E) (Fig 4). The results in our study provided insights into the different modulations of β2 subunit between mammalian and *Drosophila* Slo1/β2 channels and structural basis underlie the activation and pre-inactivation of other BK(β) complexes.

## Materials and Methods

### Constructs and mutations

To construct dSlo1-EGFP, EGFP in pEGFP-N1 (Clontech) was cut by two restriction enzymes and subcloned into pcDNA3.1(+) (Invitrogen). Next, dSlo1 was fused in-frame to the N-terminus of EGFP using appropriate restriction enzymes. The hβ2-72Myc construct was created by adding the sequence of Myc (EQKLISEEDL) between positions 72 and 73 in hβ2 via sequential overlap extension PCR and it was then subcloned into pcDNA3.1. Truncations of the β2 subunit, i.e., ΔFIW-hβ2 and Δ30-hβ2, were generated by removing amino acids from positions 2–4 and from positions 2–31, respectively. The mutations were introduced using a QuickChange Site-Directed Mutagenesis Kit (Stratagene). All of the constructs and point mutations were verified by direct DNA sequence analysis. Fig 1 shows topological maps of the constructs and mutations used in all of the experiments.

### Immunofluorescence and confocal microscopy

At about 24 h after transfection, cells were fixed with 2% paraformaldehyde in phosphate-buffered saline (PBS) for 10 min. After blocking with 2% bovine serum albumin (BSA) for 30 min, cells were incubated with mouse monoclonal anti-human Myc antibody (Abcam) at a dilution of 1:300 in 1% BSA (Abcam) for 3 h at room temperature (RT). The cells were then washed with PBS and incubated with rhodamine-conjugated goat anti-mouse IgG (H + L) (1:300) (Proteintech) for 1.5 h at RT. Nonspecific secondary antibodies were removed by washing with PBS, followed by a final soak in PBS. Cells were visualized by confocal laser scanning microscopy (Olympus IX71) using a 100× oil immersion lens (NA1.30). Parameter selection, sample scanning, and image acquisition were all controlled using Andor IQ 2 software.

### Image analysis and statistics

In experiments with coexpressed dSlo1-EGFP (green) and hβ2-72Myc (red), four cases were observed: no fluorescence, red fringes, green cytoplasm, and green cytoplasm with red fringes. In the statistical analysis, we ignored cells with no fluorescence or those only with red fringes due to the non-expression or expression of only hβ2-72Myc in HEK293 cells. The fluorescent intensity of the red fringes was calculated using ImageJ 1.45 (Wayne Rasband, National Institutes of Health) and normalized against the intensity of the control group. The representative images shown in the figures were obtained with AutoQuantX2. The error bars represent the standard error of the mean (SEM).

### Cell culture and transient transfection in HEK293 cells

HEK293 cells were cultured in Dulbecco’s modified Eagle's medium (Gibco) supplemented with 10% fetal bovine serum (Gibco) and 100 U/ml penicillin and streptomycin in incubators at 37°C under 5% CO_2_. Cells were transferred to 24-well plates before transfection and then co-transfected with α and β subunits, and the reporter gene EGFP at a molar ratio of 1:4:1 for 4–6 h, and then transiently transfected using Lipofectamine 2000 (Invitrogen) according to the manufacturer's protocol. Cells were transferred to poly-D-lysine (Sigma) coated chambers for imaging or to prepare slides for electrophysiological experiments.

### Patch clamp recording

All of the experiments were performed using excised patches in an inside-out recording configuration. Transfected HEK293 cells were prepared at 24 h after transfection in 160 K^+^ solution containing (in mM): 160 MeSO_3_K, 2 MgCl_2_, 10 HEPES (pH 7.0). Patch pipettes were pulled from borosilicate glass capillaries with resistances of 2–4 megohms when filled with the pipette solution. Experiments were performed using a PC2C patch-clamp amplifier and its related software (InBio China), where the currents were typically digitized at 100 kHz and filtered at 5 kHz. For single channel recordings, experiments were performed using an EPC-9 patch-clamp amplifier and its related software (HEKA, Germany). The currents were digitized at 50 kHz and filtered at 15.7 kHz to reduce the impact of the filter settings [70]. During inside-out recordings, solutions with different Ca^2+^ concentrations were applied to the membrane patches using a perfusion pipette containing eight solution channels. All of the experiments were performed at RT (22–24°C).

### Data analysis

Macropatch recordings were analyzed using IGOR (Wavemetrics, Lake Oswego, OR), Clampfit (Axon Instrument, Inc.), and Sigmaplot (SPSS. Inc.). Unless stated otherwise, the data are expressed as the mean ± standard deviation (SD). The activation and inactivation time constants of the currents were fitted by a single exponential equation: I = a*exp(-(t/τ)) + c, where τ is the time constant, a is the current amplitude, and c is the current offset constant. Single-channel recording data were analyzed using QuB (SUNY at Buffalo). Histograms were fitted by double or triple Gaussian equations: f = a*exp(-(x-b)^2/c) + e*exp(-(x-g)^2/h) + i*exp(-(x-j)^2/k), where a, e and i are count numbers, b, g and j are means, c, h and k are deviations.

## Supporting Information

S1 Fig Fused fluorescent proteins (-GFP and Myc-) did not alter the gating properties of WT channels. Macroscopic currents of dSlo1-GFP (green) and dSlo1/β2-72Myc (red) channel in the presence of 10 μM Ca^2+^.
(TIF)

S2 Fig β2(K33E,R34D,K35E) expressed well in dSlo1/β2(K33E,R34D,K35E) channel. Macroscopic currents of dSlo1 (left) and dSlo1/β2(K33E,R34D,K35E) (right) channel in the presence of 10 μM Ca^2+^. The amplitude of dSlo1/β2(K33E,R34D,K35E) was much smaller than dSlo1 alone.
(TIF)

S3 Fig Macroscopic currents recorded at 100 mV in 10 μM Ca^2+^. Macroscopic currents of mSlo1/β2(W4E), mSlo1/β2(W4E,D16R,E17K), dSlo1, dSlo1/β2, dSlo1/Δ3-β2, and dSlo1/Δ30-β2 recorded at 10 μM Ca^2+^, the green lines were currents recorded at 100 mV and fitted by single exponential function (red lines).
(TIF)

S4 Fig C-Linker and AC region of Slo1 channel. (A) Left: sequence alignment of dSlo1, mSlo1, and hSlo1 C-linker and AC regions. The secondary structures of the C-linker and AC region are indicated. Conserved residues are shaded at two levels (black and gray). The different positively charged residues between mammalian and *Drosophila* Slo1 were labeled in red. Right: structure and location of the C-linker and AC region (green) in the Slo1 channel, with other parts of the Slo1 channel (transmembrane domain and RCK domain) shown in gray.
(TIF)
