# Supplementary figures and images for: Single Channel Recordings Reveal Differential β2 Subunit Modulations Between Mammalian and Drosophila BKCa(β2) Channels
Source: PLoS One. 2016 Oct 18;11(10):e0163308. doi: 10.1371/journal.pone.0163308 (PMC5068790; doi:10.1371/journal.pone.0163308)

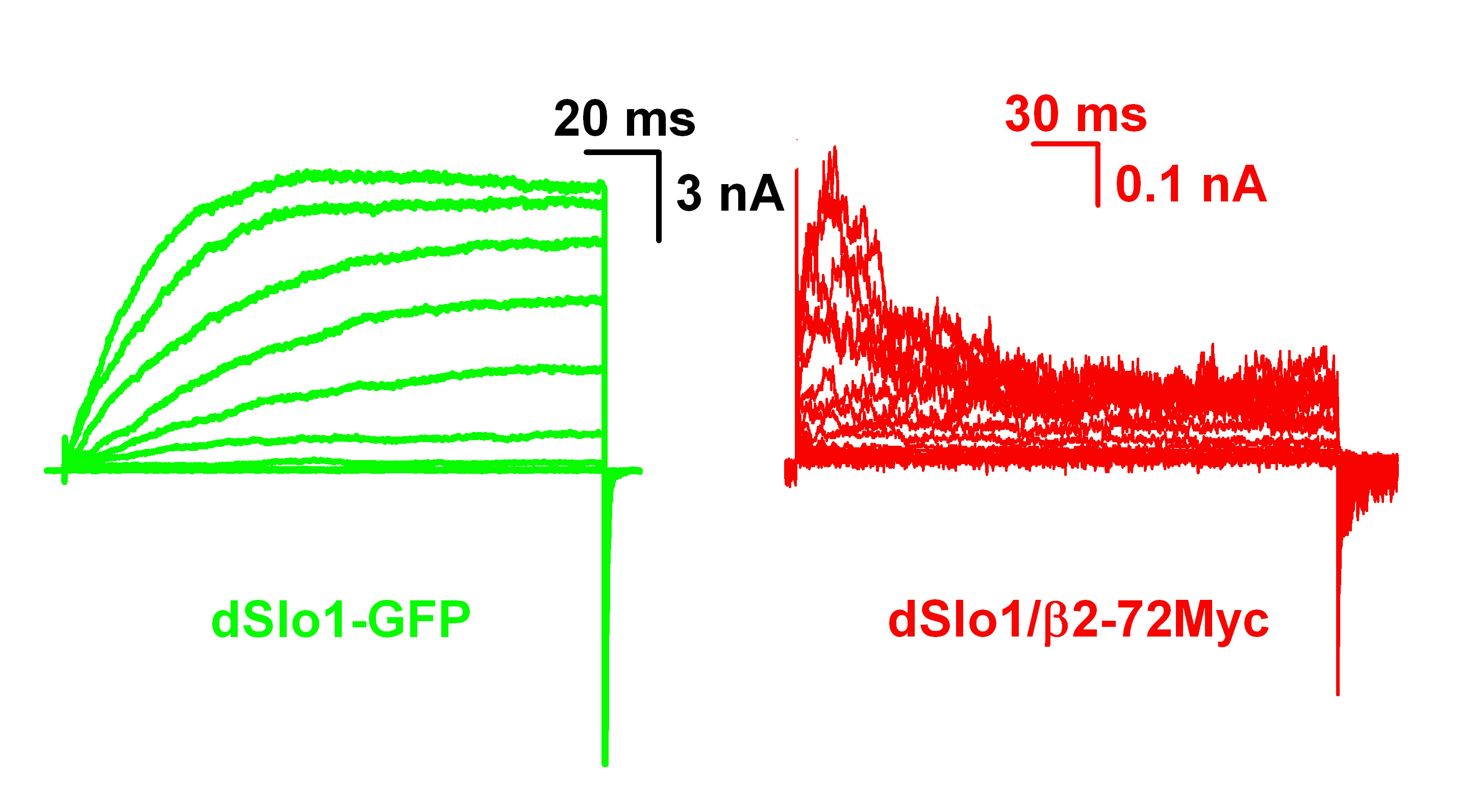

Supplement: S1 Fig — Macroscopic currents of dSlo1-GFP (green) and dSlo1/β2-72Myc (red) channel in the presence of 10 μM Ca2+. (TIF) [file pone.0163308.s001.tif]

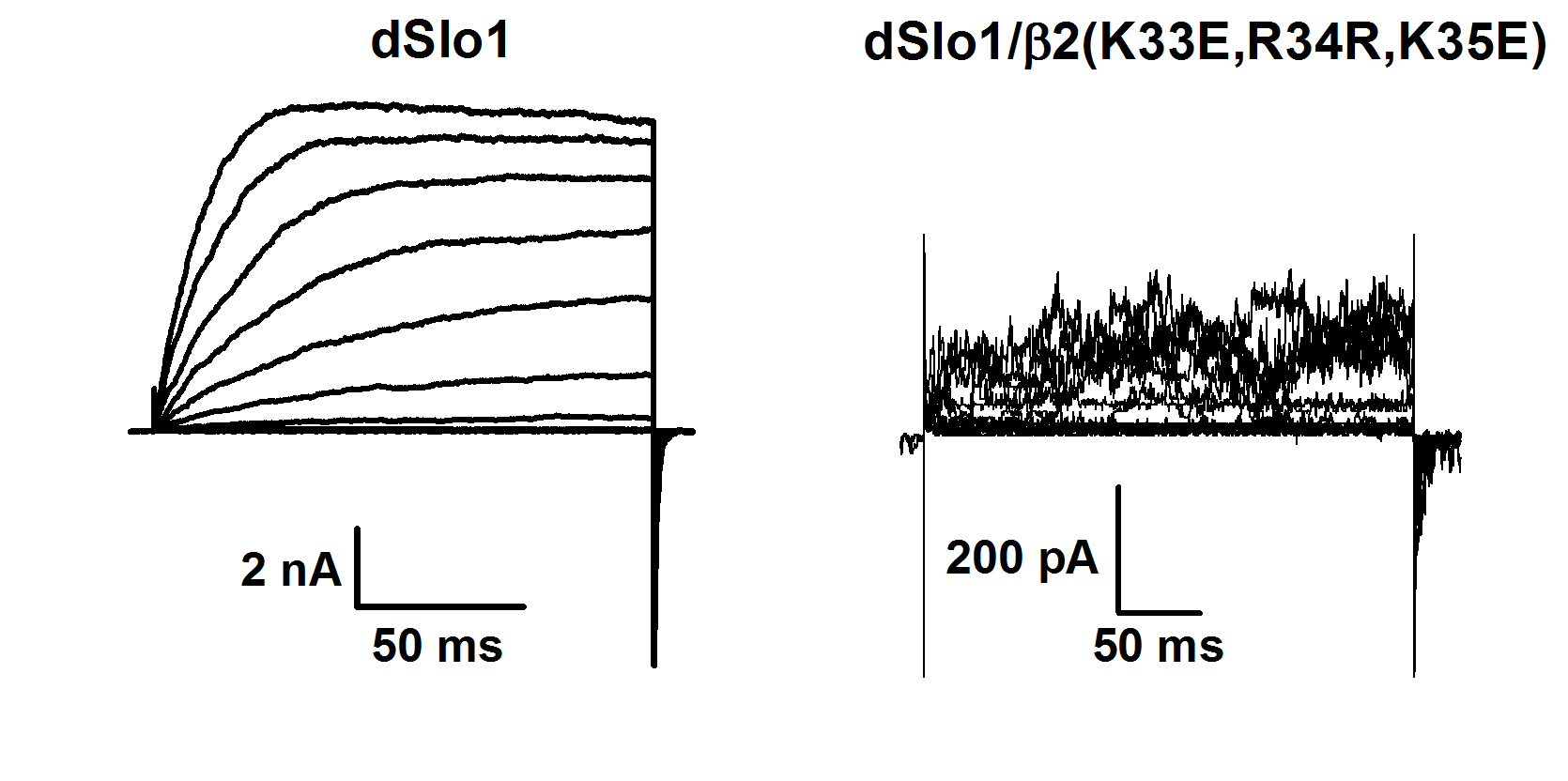

Supplement: S2 Fig — Macroscopic currents of dSlo1 (left) and dSlo1/β2(K33E,R34D,K35E) (right) channel in the presence of 10 μM Ca2+. The amplitude of dSlo1/β2(K33E,R34D,K35E) was much smaller than dSlo1 alone. (TIF) [file pone.0163308.s002.tif]

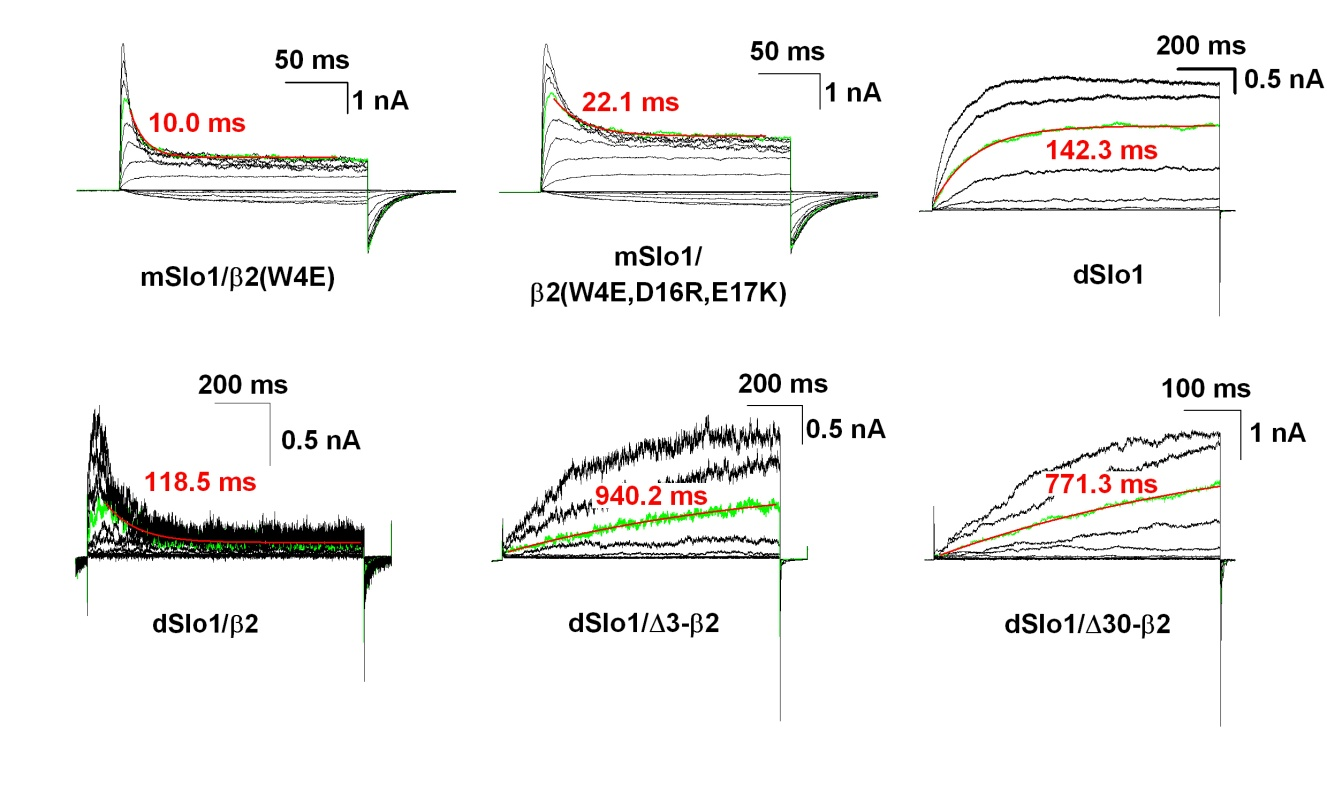

Supplement: S3 Fig — Macroscopic currents of mSlo1/β2(W4E), mSlo1/β2(W4E,D16R,E17K), dSlo1, dSlo1/β2, dSlo1/Δ3-β2, and dSlo1/Δ30-β2 recorded at 10 μM Ca2+, the green lines were currents recorded at 100 mV and fitted by single exponential function (red lines). (TIF) [file pone.0163308.s003.tif]

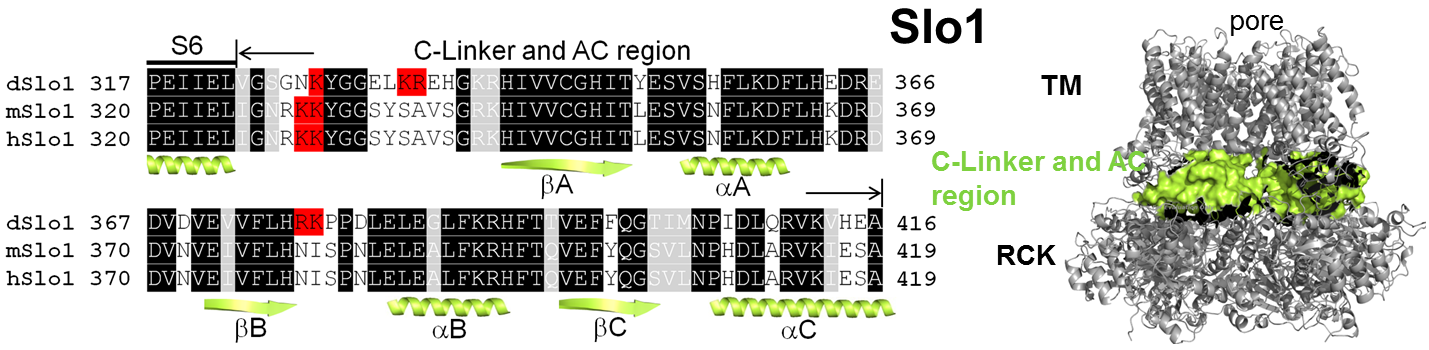

Supplement: S4 Fig — (A) Left: sequence alignment of dSlo1, mSlo1, and hSlo1 C-linker and AC regions. The secondary structures of the C-linker and AC region are indicated. Conserved residues are shaded at two levels (black and gray). The different positively charged residues between mammalian and Drosophila Slo1 were labeled in red. Right: structure and location of the C-linker and AC region (green) in the Slo1 channel, with other parts of the Slo1 channel (transmembrane domain and RCK domain) shown in gray. (TIF) [file pone.0163308.s004.tif]
